# A Western dietary pattern is associated with overweight and obesity in a national sample of Lebanese adolescents (13–19 years): a cross-sectional study

**DOI:** 10.1017/S0007114515003657

**Published:** 2015-10-02

**Authors:** Farah Naja, Nahla Hwalla, Leila Itani, Sabine Karam, Abla Mehio Sibai, Lara Nasreddine

**Affiliations:** 1 Nutrition and Food Sciences Department, Faculty of Agriculture and Food Sciences, American University of Beirut, PO Box 11-0236 Riad El Solh 11072020 Beirut, Lebanon; 2 Department of Nutrition & Dietetics, Faculty of Health Sciences, Beirut Arab University, PO Box 11-5020 Riad El Solh 11072809 Beirut, Lebanon; 3 Department of Epidemiology and Population Health, Faculty of Health Sciences, American University of Beirut, PO Box 11-0236 Riad El Solh 11072020 Beirut, Lebanon

**Keywords:** Dietary patterns, Obesity, Overweight, Adolescents, Lebanon

## Abstract

Adolescent obesity is associated with both immediate and longer-term health implications. This study aims to identify dietary patterns among a nationally representative sample of Lebanese adolescents aged between 13 and 19 years (*n* 446) and to assess the association of these patterns with overweight and obesity. Through face-to-face interviews, socio-demographic, lifestyle and anthropometric variables were collected. Dietary intake was assessed using a sixty-one-item FFQ. Dietary patterns were derived by factor analysis. The following two dietary patterns were identified: Western and traditional Lebanese. The Western pattern was characterised by high consumption of red meat, eggs and fast-food sandwiches. The traditional Lebanese pattern reflected high intakes of fruits and vegetables, legumes and fish. Female sex and a higher maternal education level were associated with a greater adherence to the traditional Lebanese pattern. As for the Western pattern, the scores were negatively associated with crowding index, physical activity and frequency of breakfast consumption. After adjustment, subjects belonging to the 3rd tertile of the Western pattern scores had significantly higher odds of overweight compared with those belonging to the 1st tertile (OR 2·3; 95 % CI 1·12, 4·73). In conclusion, two distinct dietary patterns were identified among adolescents in Lebanon: the traditional Lebanese and the Western, with the latter pattern being associated with an increased risk of overweight. The findings of this study may be used to guide the development of evidence-based preventive nutrition interventions to curb the obesity epidemic in this age group.

The Eastern Mediterranean region is currently witnessing one of the highest burdens of childhood and adolescent obesity worldwide^(^ ^1^ ^)^. Recognised as a period of rapid body growth and development with concomitant changes in body composition, insulin sensitivity and adipokine levels, as well as dietary habits and physical activity patterns, the adolescent years represent a critical stage for the development of obesity^(^ ^2^ ^,^ ^3^ ^)^.

Adolescent obesity is associated with both immediate and longer-term health implications^(^ ^4^ ^)^. Among the immediate risks are metabolic abnormalities including increased blood cholesterol, TAG and glucose levels, insulin resistance and hypertension^(^ ^2^ ^,^ ^5^ ^)^. As for the long-term health risks, studies have consistently demonstrated that, compared with obesity in early childhood, obesity in adolescence is a stronger predictor of adult obesity^(^ ^6^ ^)^. Although an obese 4-year-old child has a 20 % chance of becoming an obese adult, the likelihood that obesity in adolescence would persist into adulthood has increased to 80 %^(^ ^7^ ^)^. Adolescent obesity was also reported to predict a broad range of adverse health effects in adulthood, including type 2 diabetes, CVD and certain types of cancer, in addition to psychological disturbances^(^ ^7^ ^,^ ^8^ ^)^.

Although increasing attention is being paid to the genetic contribution to obesity, the vast majority of studies have examined and reported on the association between increased adiposity and environmental and behavioural factors, including diet, lifestyle and physical activity^(^ ^2^ ^)^. However, studies focusing on the diet as a risk factor for obesity have not provided consistent evidence about the association between dietary factors and obesity in this age group^(^ ^9^ ^,^ ^10^ ^)^. This may be partly due to the use of traditional methods in nutritional epidemiology, where most of the studies investigating the association between diet and obesity have focused on the intake of individual nutrients, foods or food groups^(^ ^9^ ^,^ ^10^ ^)^. In fact, given the complexity of nutrient interactions, there has been a growing concern that the overall pattern of dietary intake should be considered when investigating relationships between nutrition and chronic conditions such as obesity^(^ ^11^ ^)^. In this context, and to overcome the limitations of the traditional methods of examining single foods or nutrients, dietary pattern analysis was proposed as an approach that examines the joint effects of multiple dietary components on obesity^(^ ^9^ ^,^ ^12^ ^)^. Despite some inconsistencies, it has been suggested that Western dietary patterns, characterised by high-sugar and high-fat foods, are associated with higher obesity risk in adolescents, whereas healthier dietary patterns rich in whole grains, legumes and vegetables have favourable effects on BMI in this age group^(^ ^9^ ^,^ ^13^ ^–^ ^16^ ^)^. It is important to note that these studies have been mostly conducted in high-income countries and as such these findings may not be directly applicable to low- and middle-income countries, given the context-specific nature of dietary patterns^(^ ^9^ ^,^ ^13^ ^–^ ^16^ ^)^.

In Lebanon, recent studies have suggested a worrisome increasing secular trend in the prevalence of obesity among adolescents. When comparing data provided by national surveys conducted in 1997 and 2009, an approximate 2-fold increase in the prevalence of obesity among 10–19-year-old Lebanese adolescents was noted (5·9 % in 1997 *v*. 10·2 % in 2009)^(^ ^17^ ^)^. The high rate of obesity among Lebanese youth coupled with its increasing secular trend highlights the need for rigorous investigations of its determinants and associated factors^(^ ^18^ ^)^. In this context, and based on data stemming from a nationally representative survey in Lebanon, this study aimed to (i) identify and characterise dietary patterns among Lebanese adolescents using factor analysis; (ii) investigate the association of these patterns with socio-demographic and lifestyle characteristics; and (iii) evaluate the association of these patterns with overweight and obesity as assessed by BMI *z*-scores. By providing further insight into diet–obesity associations in youth, the findings of this study could catalyse the development of effective interventions and policies aimed at curbing the obesity epidemic in Lebanon.

## Methods

This study was based on a secondary analysis of data from the National Nutrition and Non-Communicable Disease Risk Factor Survey conducted in Lebanon between the years 2008 and 2009. This survey was carried out on a nationally representative sample of Lebanese adults, adolescents and children aged 6 years and above between May 2008 and August 2009. Households, the primary sampling units in this survey, were drawn using a stratified cluster random sampling frame. The strata were the six administrative Lebanese governorates, whereas the clusters were selected further at the level of districts. Using the household roster, one adult from each household and one child/adolescent from every other household were selected. The distribution of the study sample by sex and 5-year age group was similar to that of the Lebanese population as estimated by the Central Administration for Statistics in Lebanon^(^ ^19^ ^)^. Sample size needed from each age group was calculated based on previous prevalence rates of obesity as obtained from a national survey conducted in Lebanon in 1997^(^ ^20^ ^)^, using a power of 80 % and a 5 % margin of error. Accordingly, a total of 3636 subjects aged 6 years and above were recruited. Refusal rate at the level of the household was 10·7 %, with main reasons for refusal being lack of time or disinterest in the study. In face-to-face interviews at the participants’ house, trained nutritionists collected data from study subjects using age-specific multi-component questionnaires covering information on demographic, socio-economic and lifestyle characteristics. Dietary intake was assessed using a semi-quantitative FFQ. All the questionnaires used for data collection were designed by a panel of experts, including scientists in the fields of epidemiology and nutrition, and were tested on a convenience sample to check for clarity and cultural sensitivity. In addition, anthropometric measurements were obtained. Further details about the protocol and data collection procedures are described in detail elsewhere^(^ ^18^ ^)^. The design and conduct of the survey were performed according to the guidelines laid down in the Declaration of Helsinki, and all the procedures involving human subjects/patients were approved by the Institutional Review Board of the American University of Beirut. Informed consent from adults/parents and informed assent from children and adolescents were obtained before participation.

### Data collection

Of the 3636 survey participants, 446 were between 13 and 19·9 years of age, and their data were used in this study. These data included socio-demographic and lifestyle characteristics, eating habits, anthropometric measurements as well as assessment of dietary intake. Socio-demographic characteristics consisted of the following variables: age, sex, schooling level, household crowding index (obtained from the adult questionnaire), mother’s education level and working status, and parental obesity (either mother or father). Smoking status and physical activity were also considered. No questions regarding alcohol consumption were addressed to adolescents, given the cultural sensitivity of drinking in this age group and the fact that the interview took place in the presence of the parent. Physical activity assessment was carried out using a questionnaire enquiring about the weekly frequency of various activities taking place outside the school setting, including moderate-intensity activities such as playground activities, brisk walking, dancing and bicycle riding as well as higher-intensity activities such as ball games, jumping rope, active games involving running and chasing and swimming. Based on the weekly frequency, study participants were categorised into three levels of physical activity: low (never), moderate (1–2 times/week) and high (>2 times/week). Eating habits examined consisted of weekly frequencies of eating breakfast, eating out, eating while watching the television (TV) as well as the daily frequency of snack consumption.

Anthropometric measurements included weight and height. These measurements were taken using standardised techniques and calibrated equipment. Subjects were weighed to the nearest 0·1 kg in light indoor clothing and with bare feet or stockings. Using a stadiometer, height was measured without shoes. Two readings were obtained for each of the measurements and the average was used in the analysis. In this study, overweight and obesity were assessed using the BMI, calculated by dividing weight (kg) by height squared (m^2^). Sex- and age-specific BMI *z*-scores were calculated using WHO AnthroPlus software (WHO). Overweight and obesity corresponded to +1 and +2 BMI *z*-scores, respectively^(^ ^21^ ^)^.

Dietary intake of study participants was assessed using two methods: a single multiple-pass 24-h recall and a semi-quantitative FFQ. For the purpose of this study, dietary intake data obtained from the FFQ were used, given that such data are more reflective of long-term intake and result in a more valid assessment of dietary patterns. The FFQ referred to dietary intake of the previous year and included sixty-one food and beverage items commonly consumed in Lebanon. Participants were asked to record the frequency of consumption either per day, per week, per month, per year or never. This partly open-ended approach for recording frequency of consumption allows more flexibility compared with the multiple-choice frequency approach, and contributes to the reduction of misclassification errors^(^ ^22^ ^)^. Participants had the choice to report their intake either in terms of reference portion size or in grams. A reference portion, representing one standard serving expressed in household measures, was defined for each food item. Common household measures used were measuring cups, spoons, in addition to real portion size photographs. The reported frequency of each food item and beverage was then converted to a daily portion intake. The daily energy and macronutrient consumption of participants was computed using the food composition database of Nutritionist IV software and the food composition table of Middle-Eastern foods for local and traditional foods^(^ ^23^ ^,^ ^24^ ^)^.

### Derivation of dietary patterns

For the purpose of determination of dietary patterns, food items were grouped into nineteen food groups based on similarities in ingredients, nutrient profile and/or culinary usage (Table A1). Food items having a unique composition (bulgur (parboiled wheat), mayonnaise and butter/ghee) were classified individually. In addition, mayonnaise and butter/ghee were entered as separate foods, as their consumption might be indicative of a certain dietary pattern of interest. Mayonnaise is not part of the traditional Lebanese cuisine and its consumption could reflect adherence to alternate patterns. Butter and ghee are the main sources of animal fat, which could be used in cooking, and together these food items are useful in contrast with the vegetable oil cooking-based pattern. For the nineteen food groups, the total consumption was determined by summing up the daily portion intake of each item in this group. Using the dietary intake of the nineteen food groups, dietary patterns were identified by factor analysis. The latter is a data-driven method, which identifies foods that are frequently consumed together by pooling food items based on the degree to which the amounts eaten are correlated together. Its main purpose is to identify groups of food items that account for the largest variation in overall dietary intake among individuals^(^ ^25^ ^)^. Input variables in the factor analysis were adjusted in terms of their per cent contribution to total energy intake. As diagnostics for the factor analysis, the correlation matrix between the nineteen food groups was visually and statistically examined. This matrix showed a significant *χ* ^2^ (*P*<0·05) for the Bartlett’s test of sphericity and a Kaiser–Meyer–Olkin test>0·6, indicating that the correlation among the variables was sufficiently strong for a factor analysis. Furthermore, most of the correlation coefficients in the matrix were >0·3. The number of factors retained was based on three criteria: (1) the Kaiser criterion (eigenvalues >1), (2) inflection point of the scree plot (retaining factors above the elbow or break in the plot) and (3) the interpretability of factors^(^ ^26^ ^)^. The factors were rotated by a varimax rotation, in which the axes are maintained at 90°. This rotation was performed to simplify the factor matrix and to make data interpretation easier, generating non-related factors that can be used for later multiple analyses. Factor loadings indicated the strength and direction of the association between the patterns and food groups. Food groups with positive loadings contributed to the dietary pattern, whereas those with negative loadings were inversely associated with the dietary pattern. The derived dietary patterns were labelled based on food groups having a rotated factor loading >0·3^(^ ^27^ ^)^. When a food group loaded high (>0·3) on more than one factor, the factor with the highest loading was considered for factor labelling. Factor scores were calculated by multiple regression approach, and each individual received a factor score for each dietary pattern. These scores indicated the degree to which each subject’s diet corresponded to the identified pattern. For each pattern, participants were grouped into tertiles of pattern scores. In addition to the patterns derived by factor analysis, simplified dietary pattern scores were calculated for each derived pattern as the sum of standardised food variables loading high on this pattern (>0·3), following the method proposed by Schulze *et al*.^(^ ^28^ ^)^.

### Statistical analyses

Socio-demographic, lifestyle characteristics, eating habits and anthropometric measurements were described using means and standard deviations and proportions for continuous and categorical variables, respectively. Pearson’s correlation coefficients were used to examine the association of the identified dietary patterns’ scores with energy and energy-adjusted nutrient intake. Energy adjustments were carried using the residual method described by Willett *et al.* ^(^ ^29^ ^)^. Correlation coefficients were compared using Steiger-based equations^(^ ^30^ ^)^. Multiple linear regression analyses were used to examine correlates of dietary patterns in the study population. The dependent variables in these regression models were the factor scores of the identified patterns, whereas independent variables included socio-demographic and lifestyle characteristics as well as eating habits. Tests for linearity (tolerance>0·4) of the covariates included in the regression models were performed. Normality of the residuals was assessed using the histogram of standardised residuals and normal probability plot in all regression models. The associations of dietary patterns with overweight and obesity were evaluated by means of multivariate logistic regression models, with tertiles of dietary patterns’ scores as independent variables and the overweight and obesity indices as outcome variables. The Statistical Package for the Social Sciences (version 22.0^(^ ^31^ ^)^) was used for all computations, and a *P* value<0·05 was considered to be significant.

## Results

The sample of adolescents considered for this study included 226 male and 220 female participants. The distribution of this sample across the six administrative governorates of Lebanon was similar to that reported by the Central Administration of Statistics (CAS) (7·4 % Beirut, 35·9 % Mount Lebanon, 27·0 % North Lebanon, 13·8 % Bekaa, 10·1 % South Lebanon and 5·8 % Nabatiyeh)^(^ ^19^ ^)^. Furthermore, the ratio of males:females in this study was also similar to that of the CAS (1·02 *v.* 1·12). Socio-demographic and lifestyle characteristics, eating habits and anthropometric measurements of the study participants are presented in Table 1. The mean age of the study participants was 16·41 (sd 1·94) years. Over half of the mothers (59 %) had a low education level with only 16·3 % reaching university. In all, 30 % of the mothers were working. Only 7·4 % of the study participants reported smoking, with a higher percentage among males compared with females (11·9 *v*. 2·7 %; *P*<0·05). Of the surveyed adolescents, 42·8 % belonged to the ‘low’ physical activity category. The distribution of the sample across the three levels of physical activity was significantly different between males and females, where more males than females seemed to belong to the ‘high’ level (37·9 *v*. 21·9 %, *P*<0·05). On average, study participants consumed breakfast between 5 and 6 times/week (5·47 (sd 2·40)) and had a couple of snacks during the day (2·10 (sd 1·13)). The weekly frequencies of ‘eating while watching TV’ and ‘eating out’ were 4·13 (sd 3·10) and 2·19 (sd 1·72), respectively. Compared with females, male participants reported significantly higher frequencies of eating breakfast and eating out. Regarding anthropometric measurements, 10 % of the study participants were obese (BMI *z*-score≥2) and 31·2 % were overweight (BMI *z*-score≥1), with significantly higher prevalence rates being noted among boys compared with girls (Table 1).

**Table 1:** Socio-demographic and lifestyle characteristics, eating habits and anthropometric measurements of a nationally representative sample of Lebanese adolescents (Mean values and standard deviations for continuous variables; absolute and relative frequencies for categorical variables; *n* 446)

	Total		Boys (*n* 226)		Girls (*n* 220)		
	Mean	sd	Mean	sd	Mean	sd	Significance*
Socio-demographic and lifestyle characteristics							
Age (years)	16·41	1·94	16·54	2·01	16·27	1·85	*P*>0·05
Crowding index†	1·46	0·72	1·41	0·70	1·52	0·74	*P*>0·05
Current schooling level (%)							*χ* ^2^=1·66, *P*>0·05
Elementary and middle	180	40·4	86	38·1	94	42·9	
High school and vocational	217	48·8	117	51·8	100	45·7	
University	48	10·8	23	10·2	25	11·4	
Mother’s educational level (%)							*χ* ^2^=2·08, *P*>0·05
Illiterate and elementary	253	59·0	120	55·81	133	62·1	
Middle and high school	106	24·7	59	27·4	47	22·0	
University	70	16·3	36	16·7	34	15·9	
Working status of the mother (%)							*χ* ^2^=1·31, *P*>0·05
No	309	69·3	151	66·8	158	71·8	
Yes	137	30·7	75	33·2	62	28·2	
Parental obesity (%)							*χ* ^2^=1·56, *P*>0·05
No	223	73·6	99	70·2	124	76·5	
Yes	80	26·4	42	29·8	38	23·5	
Smoking status (%)							*χ* ^2^=13·83, *P*<0·05
No	413	92·6	199	88·1	214	97·3	
Yes	33	7·4	27	11·9	6	2·7	
Physical activity (%)							*χ* ^2^=14·53, *P*<0·05
Low	172	42·8	72	35	100	51	
Medium	109	27·1	56	27·2	53	27	
High	121	30·1	78	37·9	43	21·9	
Eating habits							
Breakfast frequency per week	5·47	2·40	5·74	2·26	5·18	2·51	*P*<0·05
Snacking frequency per day	2·10	1·13	2·18	1·24	2·02	1·01	*P*>0·05
Frequency of eating while watching TV per week	4·13	3·1	4·05	3·19	4·20	3·02	*P*>0·05
Frequency of eating out per week	2·19	1·72	2·63	2	1·76	1·23	*P*<0·05
Anthropometric measurements							
BMI (kg/m^2^)	22·73	4·34	23·44	4·77	22·0	3·71	*χ* ^2^=17·03, *P*<0·05
Normal (BMI *z*-score<1)	307	68·8	139	61·5	168	76·4	
Overweight (BMI *z*-score≥1)	139	31·2	87	38·5	52	23·6	
Obese (BMI *z*-score≥2)	44	9·9	34	15·0	10	4·5	

TV, television.

* *P* value was derived from the *χ* ^2^ test for categorical variables and from the independent Student’s *t* test for continuous variables.

† Crowding index was defined as the average number of people per room, excluding the kitchen and bathroom.

Factor analysis revealed two main dietary patterns, which together explained 22·6 % of the total variance in dietary intake, with the largest variance being explained by the traditional Lebanese pattern (13·19 %). Factor loadings and the variance explained by each pattern are shown in Table 2. The two patterns were called ‘traditional Lebanese’ and ‘Western’. The naming of these patterns was based on the highest food group loadings. For the traditional Lebanese pattern, the following food groups loaded higher than 0·3: vegetables, legumes, bread, rice, pasta and cereals, bulgur, fruits, fish and vegetable oils. The ‘Western’ pattern consisted mainly of poultry and eggs, red meat, mayonnaise, fast-food sandwiches, and pizza and pies. It is noteworthy that sugar-sweetened beverages had a high negative loading on the traditional Lebanese pattern (−0·54) while loading moderately on the Western pattern (0·26). In addition, milk and dairy products, although having a loading of 0·33 on the traditional Lebanese pattern, were least associated with the Western pattern (−0·51) (Table 2). A strong association was found between the traditional Lebanese and the Western patterns with their corresponding simplified patterns (*r* 0·95 for the traditional Lebanese and *r* 0·90 for the Western).

**Table 2:** Factor loading matrix of the two identified dietary patterns among a nationally representative sample of Lebanese adolescents (*n* 446)*

	Dietary patterns	
	Traditional Lebanese	Western
Vegetables	0·62†	
Sugar-sweetened beverages	−0·54	0·26
Legumes	0·52†	
Bread	0·47†	0·28
Rice, pasta and cereals	0·45†	
Bulgur	0·43†	
Fruits and fresh fruits juice	0·42†	
Fish	0·38†	0·29
Vegetable oils	0·38†	
Light soda and juice drinks	0·37†	0·23
Sweets	−0·26	
Poultry and eggs		0·63†
Milk and dairy products	0·33†	−0·51
Red meat	0·43†	0·45†
Mayonnaise		0·40†
Fast foods		0·39†
Pizza and pies		0·31†
Butter and ghee		−0·26
Fried potatoes		0·22
Per cent variance explained	13·19	8·69

* Factor loadings of <|0·2| were not listed in the table for simplicity.

† Loadings≥0·3.

The associations between the scores of the two identified patterns with energy and energy-adjusted nutrients are presented in Table 3. Although positive associations were observed between energy intake and both the patterns, the Western pattern had a significantly higher correlation (*r* 0·75 *v*. 0·50; *P*<0·0). Proteins and carbohydrates were more correlated with the traditional Lebanese than with the Western pattern (*r* 0·40 *v*. 0·2 and 0·15 *v.* −0·16, respectively; *P*<0·05). Fat and SFA intakes showed significant associations with the Western pattern (*r* 0·11 and 0·18; *P*<0·05). Fibre intake was positively correlated with the traditional Lebanese pattern (*r* 0·40) and negatively correlated with the Western pattern (−0·15). The traditional Lebanese pattern had higher correlations than the Western pattern for both Na and Ca intakes (0·20 *v*. −0·06 and 0·12 *v*. −0·46, respectively) (Table 3).

**Table 3:** Pearson’s correlations between pattern scores and energy and energy-adjusted nutrient intakes among a representative sample of Lebanese adolescents (*n* 446)†

	Dietary patterns	
	Traditional Lebanese	Western
Energy (kJ)	0·50**^a^	0·75**^b^
Energy (kcal)	0·50**^a^	0·75**^b^
Proteins (g)	0·41**^a^	0·20**^b^
Carbohydrates (g)	0·15**^a^	−0·16*^b^
Fat (g)	−0·04	0·11*
SFA (g)	−0·10*^a^	0·13*^b^
*n*-6 Fatty acids (g)	0·18**	0·03
Cholesterol (mg)	0·19**^a^	0·07*^b^
Fibre (g)	0·40**^a^	−0·15*^b^
Na (mg)	0·20**^a^	−0·06*^b^
Ca (mg)	0·12*^a^	−0·46**^b^

^a,b^ Values with unlike superscript letters were significantly different (*P*<0·05; using the Steiger’s *Z* formula to test the statistical significance of the difference between two dependent correlations). Significant correlation: * *P*<0·05, ** *P*<0·01.

† Residual energy-adjusted nutrient intake is used^(^ ^29^ ^)^.

Multivariate linear regression models were used to examine the independent associations between the socio-demographic and lifestyle characteristics with the scores of the identified dietary patterns. Table 4 represents the regression coefficients and the corresponding 95 % CI describing these associations. Compared with males, females were more likely to adhere to the traditional Lebanese pattern (*β* 0·48; 95 % CI 0·22, 0·73). A higher educational level of the mother was also associated with this pattern (*β* 0·21; 95 % CI 0·04, 0·38). Furthermore, negative associations were found between the scores of the traditional Lebanese pattern and the frequencies of eating while watching TV and eating out (*β* −0·04; 95 % CI −0·08, −0·004 and *β* −0·08; 95 % CI −0·16, −0·003, respectively). As for the Western pattern, its scores were associated with a lower crowding index (*β* −0·43; 95 % CI −0·73, −0·12), less physical activity (*β* −0·12; 95 % CI −0·22, −0·02) and a lower frequency of breakfast consumption (*β* −0·06; 95 % CI −0·11, −0·02). The frequency of eating out was, on the other hand, associated with a higher adherence to this pattern (*β* 0·14; 95 % CI 0·07, 0·21) (Table 4).

**Table 4:** Association of socio-demographic and lifestyle characteristics and eating habits with dietary patterns in a nationally representative sample of Lebanese adolescents (*β* Coefficients and 95 % confidence intervals; *n* 446)

	Dietary patterns			
	Traditional Lebanese		Western	
	*β*	95 % CI	*β*	95 % CI
Age (years)	0·04	−0·03, 0·10	0·04	−0·02, 0·10
Females *v*. males	0·48	0·22, 0·73	0·02	−0·22, 0·25
Crowding index	−0·08	−0·42, 0·25	−0·43	−0·73, −0·12
Education level of the mother	0·21	0·04, 0·38	0·06	−0·10, 0·22
Working status of the mother (working *v*. not working)	0·01	−0·28, 0·30	0·11	−0·15, 0·38
Parental obesity (obese *v*. not obese)	0·09	−0·15, 0·32	0·18	−0·040, 0·40
Smokers *v.* non-smokers	−0·28	−0·65, 0·09	−0·24	−0·58, 0·10
Physical activity	0·06	−0·09, 0·20	−0·12	−0·22, −0·02
Frequency of breakfast per week	0·02	−0·02, 0·07	−0·06	−0·11, −0·02
Frequency of snack per day	−0·09	−0·19, 0·01	−0·05	−0·14, 0·04
Frequency of eating while watching TV per week	−0·04	−0·08, −0·004	0·02	−0·01, 0·06
Frequency of eating out per week	−0·08	−0·16, −0·003	0·14	0·07, 0·21

TV, television.

Table 5 describes the results of the logistic regression analyses reflecting the associations between the identified patterns and the odds of overweight (BMI *z*-score≥1). Using age- and sex-adjusted as well as multivariate models, a consistent finding was that subjects belonging to the 3rd tertile of the Western pattern scores had significantly higher odds of overweight compared with those belonging to the 1st tertile (OR 2·12; 95 % CI 1·24, 3·63 and OR 2·3; 95 % CI 1·12, 4·73). No significant association was found between the traditional Lebanese pattern and the odds of overweight in the study population. No associations were found between the identified patterns with obesity (BMI *z*-score≥2) (data not shown).

**Table 5:** Association of the identified dietary patterns with overweight and obesity in the study population (Odds ratios and 95 % confidence intervals; *n* 446)

	Dietary patterns			
	Traditional Lebanese		Western	
	OR	95 % CI	OR	95 % CI
Age- and sex-adjusted model				
1st tertile	Ref.		Ref.	
2nd tertile	1·18	0·70, 1·99	1·19	0·68, 2·07
3rd tertile	1·60	0·92, 2·77	**2**·**12**	1·24, 3·63
*P*_trend_	<0·05		<0·01	
Multivariate model 2*				
1st tertile	Ref.		Ref.	
2nd tertile	1·25	0·63, 2·49	1·33	0·65, 2·73
3rd tertile	1·41	0·64, 3·13	**2**·**31**	1·12, 4·73
*P*_trend_	>0·05		<0·01	

Ref., referent values.

* This model is adjusted for age, sex, mother’s education, working status of the mother, parental obesity, smoking status, physical activity level, breakfast frequency per week, snacking frequency per day, frequency of eating while watching television per week, frequency of eating out per week, BMI and total energy intake.

## Discussion

In the present study, we report the results of the first national investigation of dietary patterns among Lebanese adolescents and their association with overweight, socio-demographic factors and lifestyle characteristics. Two major dietary patterns were identified in this population group: (1) the Western pattern and (2) the traditional Lebanese pattern. The Western pattern was characterised mainly by a high consumption of poultry and eggs, red meat, mayonnaise, fast-food sandwiches, and pizza and pies. The traditional Lebanese pattern reflected high intakes of vegetables, legumes, bread, rice, pasta and cereals, bulgur, fruits, fish and vegetable oils. Only the Western pattern was positively associated with overweight in the study sample. The percentage of variance of dietary intake explained by the aforementioned patterns was 22·6 %, which is in the range of what has been reported in other studies investigating dietary patterns among adolescents^(^ ^13^ ^,^ ^15^ ^,^ ^32^ ^)^. Furthermore, we have documented a strong association between the derived dietary patterns (Western and traditional) and their corresponding simplified patterns.

The identified ‘Western’ and ‘Lebanese traditional’ patterns are similar to those reported in previous studies conducted among adults in Lebanon^(^ ^33^ ^–^ ^36^ ^)^. It is, however, important to note that, although previous studies targeting Lebanese adults have identified a ‘prudent’ dietary pattern, characterised by the consumption of whole grains, low-fat and low-sugar food items, this pattern was not identified among adolescents in the present study. It may be that the adoption of ‘healthier’, more ‘prudent’ dietary habits and patterns increases with age, as nutrition-related knowledge, attitudes and perceptions become more established in adulthood and as concerns about health increase^(^ ^37^ ^)^.

The two patterns identified in this study are also similar to those derived among adolescents from other countries, despite the fact that the specific foods belonging to each pattern tend to vary in their respective level of contribution^(^ ^15^ ^,^ ^32^ ^,^ ^38^ ^,^ ^39^ ^)^. In these studies, the Western pattern is generally defined as a high-fat diet with processed meat, snack products, fast food, refined cereals and sweets. In some other studies, more specific derivatives of the Western pattern have been characterised such as the ‘junk food’, the ‘red meat’, the ‘high fat/high sugar’ or the ‘sweets and salty snacks’ pattern^(^ ^9^ ^,^ ^13^ ^,^ ^15^ ^)^. Other patterns that were reported for adolescents in the literature include the ‘fruits and vegetables’, the ‘vegetarian’ or specific variants stemming from these patterns^(^ ^9^ ^,^ ^13^ ^,^ ^14^ ^,^ ^32^ ^)^. This fruit- and vegetable-based pattern was not identified in the present study, given that fruits and vegetables are integral to the traditional Lebanese pattern, a pattern that was previously shown to conform to a large extent with existing definitions of the Mediterranean diet^(^ ^40^ ^)^. It remains important to note that factor analysis is a data-driven method and differences in the dietary assessment methods, the number and types of food groupings and statistical analysis techniques may explain the variability in dietary patterns across the literature^(^ ^41^ ^)^.

Interestingly, and in agreement with studies conducted among Lebanese adults, the results of the present study highlight sex differences in the adoption of the traditional Lebanese diet, with girls being significantly more likely to adhere to this pattern compared with boys. This corroborates the findings of previous studies reporting adolescent girls as being more health-conscious and followers of dietary recommendations compared with boys^(^ ^42^ ^,^ ^43^ ^)^. The results of the present study also showed that adherence to the traditional Lebanese dietary pattern among adolescents was associated with a higher educational level of the mother, which is in line with findings reported by the enKid study in Spain^(^ ^44^ ^)^. These findings highlight the role of maternal educational as one of the modulators of the family’s environment, eating habits and lifestyle, while also supporting the conclusions reported by Darmon & Drewnowski^(^ ^45^ ^)^, indicating that higher-quality diets are mainly consumed by better-educated individuals.

The study’s findings show that the Western dietary pattern was associated with an overall unhealthy lifestyle among adolescents, characterised by less physical activity, lower frequency of breakfast consumption and a higher frequency of eating out. These results are in line with those reported by Kourlaba *et al*.^(^ ^15^ ^)^ in Greece and by Craig *et al*.^(^ ^14^ ^)^ in Scotland. These observations could be due to the fact that it is more common for young people to consume sweets, snacks and fast food while watching TV and due to the food advertisements to which adolescents are usually exposed during viewing^(^ ^46^ ^)^. Alternatively, the observed associations may suggest that the clustering of behavioural risk factors, including breakfast skipping, physical inactivity and unbalanced diet^(^ ^47^ ^)^, which has been repeatedly described among adults^(^ ^40^ ^)^, is already apparent as early as the adolescent years^(^ ^48^ ^)^. This clustering of behavioural risk factors is increasingly prevalent in countries undergoing the nutrition transition, including Lebanon and other countries of the Eastern Mediterranean region^(^ ^40^ ^)^. A distinctive feature of the nutrition transition is the erosion of the traditional lifestyle, the shift towards an increasingly energy-dense dietary pattern and the adoption of sedentary mode of living. The association between Western pattern and lower crowding index, as observed in the present study, suggests a socio-economic gradient in the nutrition transition reflected by a higher adoption of westernised eating habits in the more affluent households. These results are in agreement with those reported among adolescents in Tunisia, another Eastern Mediterranean country undergoing the nutrition transition, where the diet of adolescents was all the more ‘modernised’ in more affluent households^(^ ^38^ ^)^.

In the present study, the Western pattern was found to be significantly associated with overweight in the Lebanese adolescent population. These findings are in agreement with those reported among 15–19-year-old Tunisian adolescents, where the ‘modern’ pattern was found to be significantly associated with overweight in boys^(^ ^38^ ^)^. Similar findings were also reported by Song *et al*.^(^ ^49^ ^)^ among Korean adolescents. A recent systematic review concluded that Western dietary patterns are positively associated with the metabolic syndrome risk factors including obesity in adolescents^(^ ^50^ ^)^. The associations of the Western dietary pattern with higher energy, fat and SFA consumption and with lower fibre intake may partially explain the relationship between adherence to this pattern and increased adiposity among adolescents. It is important, however, to note that this association was refuted by other studies^(^ ^9^ ^,^ ^13^ ^,^ ^14^ ^)^. For instance, adherence to a ‘sweet and salty snack food’ pattern was found to be protective against obesity in adolescents from the USA and similar results were documented among Australian adolescents^(^ ^13^ ^)^. These inconsistent observations could be explained by the possibility of behavioural changes among adolescents with higher BMI, under-reporting of usual intake, social desirability bias or a wider variety of unhealthy eating patterns identified in the US and Australian studies^(^ ^9^ ^,^ ^13^ ^)^.

In this study, and in agreement with previous studies conducted among Lebanese adults, the Lebanese traditional pattern was not found to be associated with overweight in adolescents. The complex nature of the traditional Lebanese pattern makes interpretation rather difficult. The Lebanese diet, as defined in our present and previous findings^(^ ^34^ ^)^, is highly loaded on fruit and vegetables, which is further corroborated by its positive association with fibre. Similarly, the traditional Lebanese diet is positively associated with Ca, a nutrient that is suggested to play a role in body weight regulation. Based on a retrospective analysis of several studies, Heaney *et al*.^(^ ^51^ ^)^ proposed that a daily increase of 300 mg of Ca, or approximately one dairy serving, was associated with a yearly reduction of approximately 1 kg of body fat in youth. With these characteristic elements of high fruits, vegetables, fibre and Ca intakes, a negative association between the traditional Lebanese dietary pattern and risk of obesity is expected. However, some energy-dense foods such as refined grains and vegetable oils were also heavily consumed in this dietary pattern and may have counteracted the positive effects that the intake of fruits and vegetables might have had on obesity. The traditional Lebanese dietary pattern was also found to be associated with Na intake, which may have further counteracted the beneficial effects that other dietary constituents may have had on adiposity. In fact, it was recently shown that high Na intakes are positively associated with adiposity among healthy adolescents, with this association being independent of total energy intake and sugar-sweetened soft drink consumption^(^ ^52^ ^)^.

The strengths of this study include the national representation of the studied population and the objective method adopted to measure height and weight. In addition, the simplified dietary patterns derived in this study would allow the use of the factor analysis-based patterns in other populations, especially in light of the observed association between the Western pattern and overweight among adolescents. The results of this study should, however, be interpreted in light of the following limitations. The cross-sectional design of the study allows us to test associations rather than to assess any causal relationship. In addition, this study relied on the use of the FFQ for dietary assessment, which may be limited by measurement errors, reliance on memory and the number of food items included in the food list^(^ ^53^ ^)^. An important source of error in the FFQ is portion size estimation. Studies have shown that untrained individuals find it challenging to estimate portion sizes of foods previously consumed. In this study, subjects were given the choice to report their intakes in terms of grams or as a function of a reference portion size. The reference portion, representing one standard serving, was expressed in household measures such as cups, spoons and plates. Real-size photographs were used to assist subjects in the identification of the reference portion size. Another limitation of the FFQ is the restriction imposed by a fixed list of foods. The food list contained only a limited number of core foods with little information about food preparation methods, foods consumed together as meals or mixed dishes, type of food, brand names, source of food, time of meals and snacks, and ingredients^(^ ^54^ ^)^. In this study, to lessen the restriction imposed by the fixed food list of the FFQ, a question about ‘other foods usually consumed’ was asked at the end of the interview. Despite these limitations, it has been shown that the FFQ is one of the most suitable dietary assessment tools in large epidemiological studies as it provides information on the subject’s habitual diet over longer periods of time^(^ ^55^ ^)^. It is important to note that, although the FFQ used in the present study was not validated in our study population, it was previously used for the assessment of dietary patterns and their relationship with obesity and the metabolic syndrome among Lebanese adults and has yielded plausible findings^(^ ^33^ ^,^ ^34^ ^)^. The FFQ used in the present study was administered by trained nutritionists rather than being self-administered. This approach provides several advantages, as self-administration of the FFQ requires a literate population, and may result in inconsistent interpretations of the food list and lower response and completion rates, each of which may jeopardise the validity of the data^(^ ^56^ ^)^. Such an interviewer-based approach, however, may introduce a potential social desirability bias, where respondents may deliberately answer questions inaccurately in a manner that will be viewed favourably by the interviewer^(^ ^57^ ^)^. In order to minimise such a bias, interviewers were trained to limit any judgemental verbal and non-verbal communication during the completion of the questionnaire. Finally, factor analysis is a data-driven method, which tends to define population-specific patterns. Therefore, the results of this study are likely to represent patterns that are, in some aspects, specific to the Lebanese adolescent population. In addition, the use of factor analysis necessitates several arbitrary assumptions pertinent to the selection of food groups, the number of retained factors and their labels^(^ ^58^ ^)^. The food groupings that were adopted were comparable with those reported by previous investigations conducted in Lebanon^(^ ^33^ ^,^ ^34^ ^,^ ^36^ ^)^ as well as by other studies worldwide^(^ ^59^ ^,^ ^60^ ^)^. As for the number of factors retained, although four had an eigenvalue>1 and were above a breaking point in the scree plot, only two factors satisfied the third criterion for the factors’ selection – interpretability of factors – and hence were retained in this study. Furthermore, although a few studies have applied similar factor loading cut-off for labelling as used in this study, ‘0·3’^(^ ^61^ ^,^ ^62^ ^)^, others have applied different cutoffs such as ‘0·5’^(^ ^63^ ^)^, ‘0·35’^(^ ^64^ ^)^ and ‘0·25’^(^ ^65^ ^)^. For consistency with our earlier investigations of dietary patterns and their association with disease in Lebanon, we have opted to use the 0·3 cut-off in this study.

### Conclusion

The present study identified two main dietary patterns among the Lebanese adolescent population: the ‘Western’ and the ‘traditional Lebanese’, with the Western pattern being associated with a higher risk of overweight in the study population. These findings lay the grounds for culture-specific interventions targeted at reducing rates of adolescent overweight and obesity, which have been shown to be on the rise in Lebanon^(^ ^17^ ^)^. Interventions aiming at discouraging the adoption of Western dietary patterns, characterised by high intake of fast food and other energy-dense foods such as pies and pizzas, fried potatoes, while promoting the consumption of the traditional Lebanese pattern based on fruit and vegetables, should be developed. In fact, the provision of dietary guidance based on a dietary pattern approach is often clearer and easier to understand and interpret compared with nutrient-based approaches, and this may be particularly true in youth^(^ ^66^ ^)^. Knowing that many of the habits and food skills acquired during adolescence are sustained later in life, interventions aimed at altering dietary behaviours in the early adolescent years have the potential of affecting the individual’s lifetime risk for diet-related diseases^(^ ^67^ ^)^.

## Appendix

[Table tabA1]

**Table A1 tabA1:** Food groupings based on culinary usage and nutrient content

Food group	Food components
Vegetables	Salad greens (lettuce, celery, green peppers and cucumbers), dark or deep yellow vegetables (spinach, hindbeh, carrots), tomatoes (fresh and cooked), green peas, squash and eggplant, cruciferous vegetables (cauliflower, cabbage and broccoli), potatoes cooked
Sugar-sweetened beverages	Bottled fruit juices, regular soft drinks
Legumes	Fava beans, chickpeas, lentils, beans, etc.
Bread	White and whole bread
Rice, pasta and cereals	Rice, pasta and breakfast cereals
Bulgur	Parboiled wheat
Fruits and fresh fruits juice	Fresh fruit juices, citrus orange (e.g. orange), deep yellow or orange (e.g. peach, apricots), strawberry, grapes, banana, apple and dried fruits (raisins, dates, apricots)
Fish	Fish fried, broiled and canned
Vegetable oils	Vegetable oil, olive oil
Light soda beverages	All types of light beverages including soda and juice drinks
Sweets	Added sugars (e.g. honey, jam, sugar, molasses), chocolate, ice cream, Arabic sweets, pastries (e.g. cakes, cookies, doughnuts, muffin, croissant)
Poultry and eggs	All types of chicken boiled, broiled, fried, stewed, breaded and eggs
Milk and dairy products	Milk, yoghurt, labneh (strained yoghurt) and cheese (light and whole milk)
Red meat	Red meat, organ meat (liver, kidney, brain), luncheon meats (mortadella, jambon, salami, turkey), sausages (makanek and hot dogs)
Mayonnaise	Mayonnaise and mayonnaise-based salad dressing
Fast food	Falafel sandwiches, chawarma sandwiches, hamburger
Butter ghee	Butter and ghee
Fried potatoes	Fried potatoes, chips
Pizza and pies	All types of pizza (manakish zaatar, cheese and pizza)
